# Quantification of capture efficiency, purity, and single-cell isolation in the recovery of circulating melanoma cells from peripheral blood by dielectrophoresis†

**DOI:** 10.1039/d2lc01113a

**Published:** 2023-04-07

**Authors:** Han Chen, Sommer Y. Osman, Devon L. Moose, Marion Vanneste, Jared L. Anderson, Michael D. Henry, Robbyn K. Anand

**Affiliations:** a Department of Chemistry, Iowa State University Ames Iowa 50011 USA rkanand@iastate.edu; b Departments of Molecular Physiology and Biophysics, University of Iowa Iowa City IA 52242 USA; c Pathology, Urology and Radiation Oncology, University of Iowa Iowa City IA 52242 USA; d Holden Comprehensive Cancer Center, University of Iowa Iowa City IA 52242 USA

## Abstract

This paper describes a dielectrophoretic method for selection of circulating melanoma cells (CMCs), which lack reliable identifying surface antigens and are extremely rare in blood. This platform captures CMCs individually by dielectrophoresis (DEP) at an array of wireless bipolar electrodes (BPEs) aligned to overlying nanoliter-scale chambers, which isolate each cell for subsequent on-chip single-cell analysis. To determine the best conditions to employ for CMC isolation in this DEP-BPE platform, the static and dynamic dielectrophoretic response of established melanoma cell lines, melanoma cells from patient-derived xenografts (PDX) and peripheral blood mononuclear cells (PBMCs) were evaluated as a function of frequency using two established DEP platforms. Further, PBMCs derived from patients with advanced melanoma were compared with those from healthy controls. The results of this evaluation reveal that each DEP method requires a distinct frequency to achieve capture of melanoma cells and that the distribution of dielectric properties of PBMCs is more broadly varied in and among patients *versus* healthy controls. Based on this evaluation, we conclude that 50 kHz provides the highest capture efficiency on our DEP-BPE platform while maintaining a low rate of capture of unwanted PBMCs. We further quantified the efficiency of single-cell capture on the DEP-BPE platform and found that the efficiency diminished beyond around 25% chamber occupancy, thereby informing the minimum array size that is required. Importantly, the capture efficiency of the DEP-BPE platform for melanoma cells when using optimized conditions matched the performance predicted by our analysis. Finally, isolation of melanoma cells from contrived (spike-in) and clinical samples on our platform using optimized conditions was demonstrated. The capture and individual isolation of CMCs, confirmed by post-capture labeling, from patient-derived samples suggests the potential of this platform for clinical application.

## Abstract 2

Dielectrophoretic capture of circulating melanoma cell (CMC) and white blood cells (WBCs) is quantified as a function of electric field frequency, and the optimized conditions are utilized to isolate CMCs from patient-derived blood samples.

## Graphical abstract

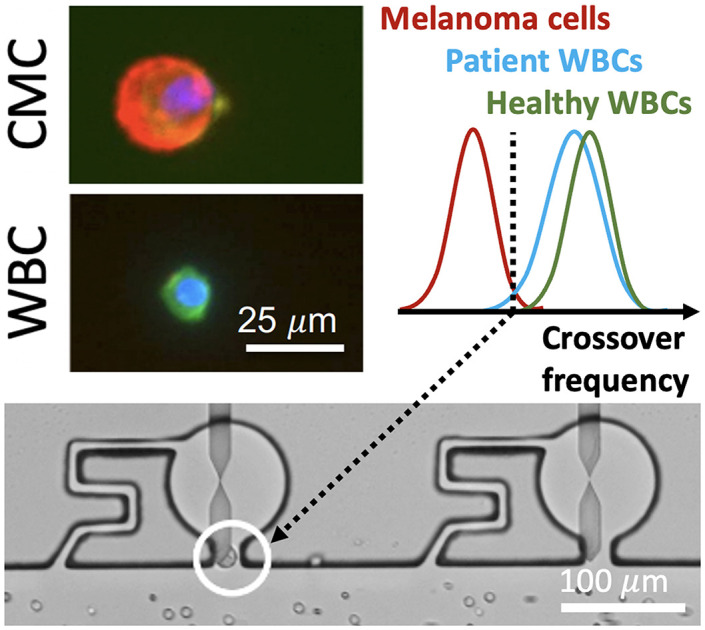

## Introduction

Metastasis is responsible for 90% of cancer deaths^1^ and occurs through the migration of cancer cells, primarily through the blood stream, from a localized tumor to new locations in the body. In the treatment of an individual patient, the number and characteristics of circulating tumor cells (CTCs) can be correlated to disease prognosis and to the likelihood of response to therapy.^2–4^ This information has the potential to allow physicians to design tailored treatment plans, thus leading to improved outcomes for patients. However, the enormous value of CTCs has not been completely realized, due to challenges in the detection and isolation of CTCs.^5^ CTCs are extremely rare (as few as 1 CTC per 1 × 10^9^ hematological cells) and possess highly heterogeneous physical and biological characteristics.^6–8^ Therefore, accurate selection of CTCs from blood cells is the primary challenge. Further complicating this challenge is the fact that, due to spatial and temporal tumor heterogeneity, meaningful information is gained by testing CTCs individually. Unlocking the clinical utility of CTCs lies in the ability to detect and isolate these rare cells using methods amenable to downstream characterization and applications.^9^ Currently, the CellSearch® system (Menarini Silicon Biosystems, Inc.) is the only FDA-approved diagnostic system for enumeration of CTCs in patients with breast, prostate, and metastatic colorectal cancers.^4,10^ Although CTC enumeration using this system provides prognostic value in cancer patients, CTCs are rendered nonviable thereby limiting downstream analysis and preventing *ex vivo* cell culture. Therefore, there is a clear need to develop technologies that facilitate viable CTC recovery following the cell enrichment stage.

While many methods exist for selective detection of CTCs, they do not provide a pure and representative sample. These techniques are either over-selective and miss certain cell populations, thus biasing results, or are under-selective and do not result in highly pure samples of CTCs. For example, immunoaffinity-based approaches are predominately targeted towards the cell-surface antigen EpCAM.^11–13^ However, EpCAM has been shown to be downregulated in CTCs during an epithelial-to-mesenchymal transition (EMT), in which these cells adopt a more mobile phenotype that confers a metastatic advantage.^14^ In melanoma, EpCAM expression is completely absent since melanocytes originate from the neural crest and not the epithelium,^15^ which magnifies the difficulty of enrichment and detection of target cells. In addition, circulating melanoma cells (CMCs) are a very heterogeneous population of cells,^16^ yet current techniques used to enrich melanoma cells from blood do not commonly consider this factor. Some multi-marker approaches aimed at improving the sensitivity of CMC capture have been used. However, they still yielded low capture efficiency (34%) in spike-in experiments.^17,18^ This means, technologies that rely on antigen expression levels of CMCs are overly specific and lose key cell populations. In contrast, methods that select for cell size alone, such as ISET® filtration, result in significant contamination by white blood cells (WBCs) and circulating nevus cells, due to the overlap between the size distributions of these cell types.^19,20^ Some other approaches, such as the CTC-iChip,^21^ which selects CTCs based on size using deterministic lateral displacement (DLD) and inertial focusing followed by negative depletion and, separately, OncoQuick®,^22^ which is based on centrifugation and filtration have both demonstrated an ability to capture CMCs. These platforms are effective at isolating rare cells from patients' blood. However, some improvements are still needed for these methods to be widely applied clinically. Most importantly, they have a limited ability to perform single-cell molecular analysis.^23^ In summary, the correct degree of selectivity is critical to obtaining an accurate picture of metastatic burden and drug resistance.

Dielectrophoresis (DEP) is a field-induced force (*F*_DEP_) acting on a polarizable particle when exposed to a non-uniform electric field (an electric field gradient). The magnitude and sign of this force is a function of the frequency of the electric field and depends on the composition and morphology of biological cells.^24^ When *F*_DEP_ is positive, the induced DEP force, displaces particles toward higher electric field strength (pDEP), while particles move toward lower electric field strength when *F*_DEP_ is negative (nDEP). The frequency above which cells transition from nDEP to pDEP response is the crossover frequency (cof).^25,26^ Importantly, the unique frequency-dependent responses of cells allow them to be separated by DEP at a field frequency and medium conductivity where disparate values of *F*_DEP_ can be achieved.

Among cell manipulation techniques, DEP has a distinct advantage in that it offers label-free selectivity that can be coupled with cell capture in a single step.^27^ This selectivity stems from biophysical properties with high biological relevance, such as glycosylation and membrane folding.^28^ Since dielectric properties arise from the composition and morphology of the cells, they are a much more specific differentiator of phenotype than size alone while not being as overly selective as a single biomarker such as EpCAM.^29^ Cell size and per-area membrane capacitance have a similar impact on cof over the ranges relevant to cancer cells and blood cells. Therefore, separation based on DEP exhibits less selection bias when compared with size- and antibody-based approaches. Further, excellent separation efficiencies have been reported. Alazzam *et al.* applied DEP *via* interdigitated comb-like electrodes to achieve 96% separation efficiency of MDA-MB-231 cells from normal blood cells.^30^ Gascoyne *et al.* employed dielectrophoretic field-flow fractionation (DEP-FFF) to isolate tumor cells with above 90% separation efficiency from the nucleated cell fraction of blood (the “buffy coat”).^31^ Their work showed that, for melanoma cells, the per-area membrane capacitances are higher (about 25 mF m^−2^*versus* 9–16 mF m^−2^ for blood cells). In addition, membrane folding leads to 1.5- to 3-fold increases in capacitance through surface area (1- to 1.8-fold for blood cells). Despite these advantages, most DEP sorting approaches are not easily interfaced with single-cell assays. Cells are not captured individually^25,32,33^ or are not fluidically isolated to prevent assay crosstalk.^34^ While DEP microwells enable single-cell confinement,^35,36^ geometric constraints have thus far limited the reaction volumes to only 56 pL, which is insufficient for certain assays such as single-cell PCR. Therefore, DEP methods that integrate selective capture and fluidic isolation for subsequent analysis are still needed.

**Scheme 1:**
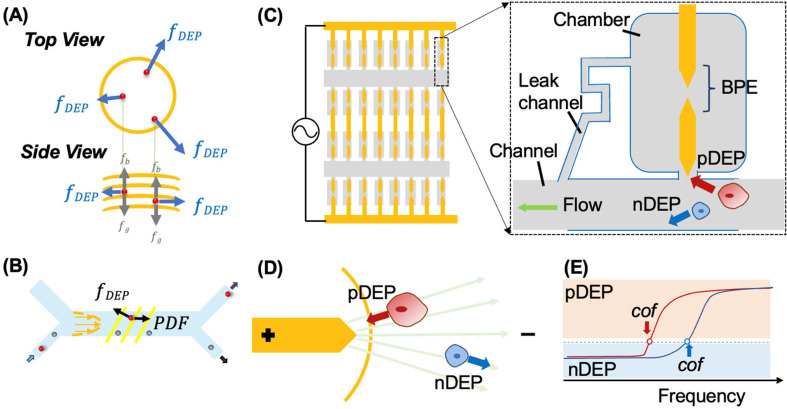
(A) and (B) illustrate the forces experienced by cells in 3DEP and CF-DEP platforms, respectively. Illustration of the DEP-BPE microfluidic device utilized for selective capture of individual CMCs (C), the principle of pDEP attraction and nDEP repulsion in a non-uniform electric field (D), and DEP spectra showing the frequency at the crossover frequency (cof) (E).

Previously, we demonstrated the use of DEP at a wireless bipolar electrode (BPE) array,^37,38^ controlled by only two electrical leads, to address the need for selective single-cell capture. This DEP-BPE device, illustrated in Scheme 1A, shows excellent single-cell capture and transfer efficiency, accomplishes selective capture, isolation, and electrical lysis of cells for subsequent assay, is scalable, and allows the assay reaction volume to be tuned.^39^ This platform is sufficiently simple to accomplish practical frequent monitoring of disease progression and therapeutic response by repeated liquid biopsies. Clinical application of such a DEP-based platform as a diagnostic tool will allow greater access to information on genotypic and phenotypic features of CTC subpopulations and thereby guide better therapeutic decisions. Specific application to melanoma is especially impactful because CMCs are highly invasive and difficult to detect and capture by traditional methods due to a lack of highly expressed biomarkers.^29^ While DEP has been reported to discriminate melanoma cells from leukocytes^26^ and even subdivide them based on melanin content,^40^ a quantitative description of separation and capture efficiencies and their dependence on applied electric field frequency and DEP methodology is needed. Further, while the response of leukocytes to DEP has been reported for healthy donors,^26^ it may differ for patients, especially those with late-stage disease. Finally, single-cell analysis is expected to be particularly informative when applied to CMCs because melanoma patients have higher than average numbers of tumor cells in their blood.^41,42^ There is therefore a specific need for the performance of DEP platforms capable of single-cell isolation to be characterized.

Here, we quantify the efficiency and selectivity of capture of CMCs by DEP at an array of wireless BPEs followed by single-cell isolation into chambers. First, to determine the best operating conditions for this DEP-BPE platform, first, the static and dynamic dielectrophoretic response of melanoma cells, patient-derived xenograft (PDX) cells and peripheral blood mononuclear cells (PBMCs) are quantified by two established DEP platforms. In this way, the selectivity of DEP separation of melanoma cells from PBMCs can be predicted at high resolution (5 kHz) as a function of frequency. The results of this evaluation reveal that each DEP method requires a distinct frequency to achieve capture of melanoma cells and that the distribution of dielectric properties of PBMCs is more broadly varied in and among patients *versus* healthy controls. These findings uncover limitations in the extrapolation of results between DEP platforms and show that blood cells from healthy donors are not representative. Based on this evaluation, we predicted that 50 kHz would provide the highest capture efficiency (99%) on our DEP-BPE platform while maintaining a low rate of capture of unwanted PBMCs (1 : 10^4^ to 1 : 10^8^). After having identified the best frequency for CMC isolation in the DEP-BPE platform, the efficiency of cell capture was measured by DEP-BPE at fixed flow rate, voltage and frequency. The capture efficiency of the DEP-BPE platform for the isolation of melanoma cells matched that predicted by our evaluation of dielectric properties of relevant cell types. Excellent single-cell capture (100%) was achieved when less than 40 melanoma cells were introduced, corresponding to occupancy of 25% of the chambers. Critically, these results inform the minimum array size that is required, in this scalable platform, to maintain high capture efficiency. Further, the isolation of melanoma cells from contrived (spike-in) and clinical samples on our platform using optimized conditions was demonstrated. The capture and individual isolation of CMCs from patient-derived samples suggests the potential of this platform for clinical application. We anticipate that the results of this quantitative study will inform the design of the next generation of DEP-based platforms for CMC isolation and analysis.

## Theory and mechanism

### Principle of manipulation of cells by DEP

DEP is the phenomenon of particle movement in response to a nonuniform electric field, which induces a dipole moment across the particle due to the electrical polarization at the particle–solution interfaces.^24–26^ The electrostatic force exerted by the non-uniform field on opposing ends of this dipole are unequal, thus resulting in translation of the particle in the field (Scheme 1B). The time averaged DEP force 
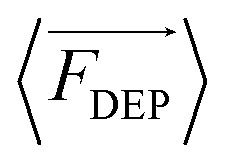
 exerted on a homogeneous spherical particle is described by eqn (1).1
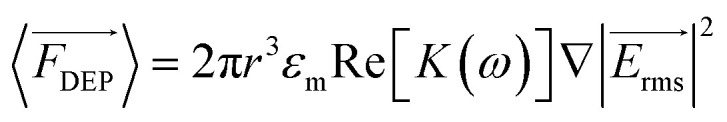


Here Re[*K*(*ω*)], *ε*_m_, *r* and *E*_rms_ are the real part of the Clausius–Mossotti (CM) factor (*K*(*ω*)), which is a function of angular frequency (*ω*), permittivity of the medium, particle radius, and the root-mean-square amplitude of the electric field, respectively. Electric field inhomogeneity is expressed by the term 
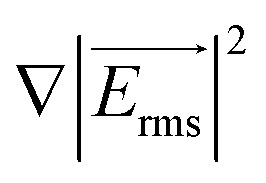
, which is dictated by the geometry and spacing of insulating structures and electrodes. The CM factor compares the complex permittivity of the particle (
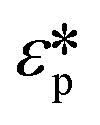
) to that of the surrounding medium (
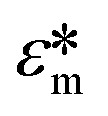
).2
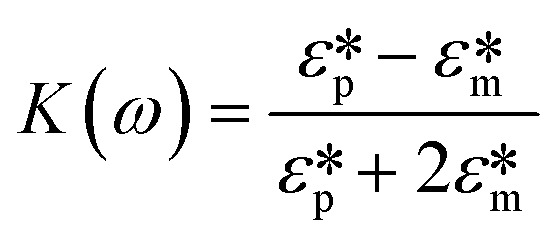


When the particle is more polarizable than the medium (Re[*K*(*ω*)] > 0), the force acts in the direction of the electric field gradient and therefore drives the particle towards increasing field strength. This scenario corresponds to positive DEP (pDEP). On the contrary, when the particle is less polarizable than the medium (Re[*K*(*ω*)] < 0), 
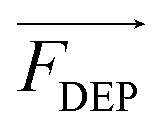
 is negative and moves the particle against the gradient, toward decreased field strength – a response which is referred to as negative DEP (nDEP). The frequency at which particles transition from nDEP to pDEP is called the crossover frequency (cof) (Scheme 1C).

The CM factor for cells is more complicated than that for a uniform spherical particle owing to their core–shell structure (membrane and cytoplasm), surface roughness (membrane folding), and for some cell types, non-spherical shape. At low electric field frequencies (below about 10 MHz), a first cof, from nDEP to pDEP is determined by exterior factors, including membrane folding and the characteristics of membrane-bound proteins and glycosylation. These features contribute to *C*_0_ – the capacitance per unit area of the cell plasma membrane, which varies substantially between different cell types.^26^ When a cell folding factor (*φ*) is introduced, the total effective capacitance (*C*_tot_) of the cell membrane is *C*_tot_ = 4π*r*^2^*φC*_0_. The value of the first cof increases with the solution conductivity (*σ*_m_). In our study, we fixed solution conductivity at 74.5 μS cm^−1^ for all the experiments, simulations, and calculations. Separation of cancer cells from blood cells can be accomplished by identifying an AC electric field frequency where the target cells experience pDEP, while non-target cells undergo zero or negative DEP.

**Scheme 2:**
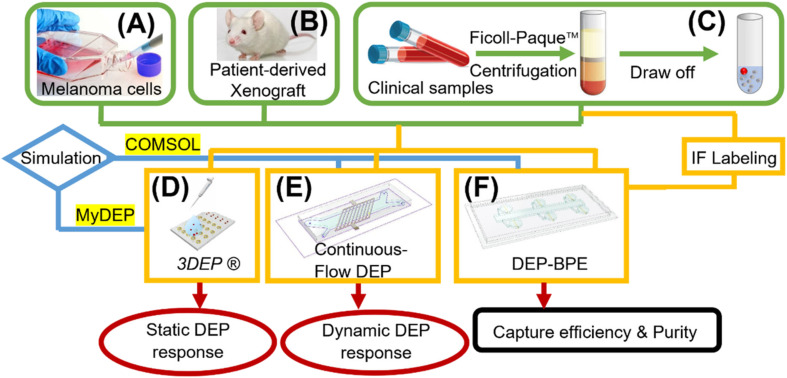
Flow diagram showing the study design used to quantify the isolation of melanoma cells from buffy coat by DEP. Three different cell sources were utilized to determine the selectivity of DEP separation of melanoma cells from PBMCs. Illustrations in (A–C) show three sources of cell examined in this study and in (D–F) show three distinct platforms utilized in experiments.

In cell separation under fluid flow, as in DEP-BPE and continuous-flow DEP (CF-DEP) platforms (Scheme 2E and F), the appropriate voltage and frequency are chosen to induce pDEP force against the hydrodynamic viscous drag force at an angle, resulting in a net force parallel to the electrodes. The viscous drag force is defined by the Stokes drag equation for a spherical cell in laminar flow as,3
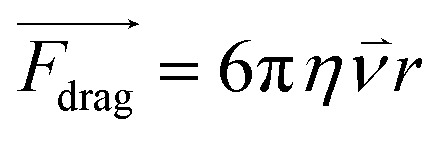


Here, *r* is the radius of the spherical object, *η* is the dynamic viscosity, and *ν⃗* is the flow velocity relative to the object. Therefore, *F*_DEP_ scales more rapidly with *r* than does *F*_drag_, and when the two are diametrically opposed, terminal velocity of the object scales with *r*^2^. This dependence makes larger cells easier to capture. The sum of the *F*_drag_ and *F*_DEP_ vectors must be a combined force that yields sufficient acceleration to displace a cell to a capture pocket (in the DEP-BPE device) or outlet for collection (in CF-DEP).

### Bipolar electrodes in microfluidic devices

A BPE is an electrical conductor in an ionically conductive phase that when exposed to an external electrical field can facilitate oxidation and reduction reactions simultaneously at its opposing ends.^43,44^ For example, a BPE can comprise a strip of metal embedded in a microfluidic channel filled with an aqueous^37^ or organic electrolyte solution.^45^ When a DC electric potential is applied between the reservoirs of the microchannel, a linear potential drop is expected along the channel length due to its high electrical resistance and assuming a uniform cross-sectional area. The linear electric field leads to potential differences between the BPE (an equipotential object) and the solution in contact with its ends.^46^ Under an AC electric potential of sufficiently high frequency, faradaic reactions are minimized, and instead, BPEs have been utilized to shape the electric field, creating local electric field maxima and minima for DEP.^38,39,47,48^ Incorporation of BPEs and BPE arrays into microfluidic devices has led to significant advancements in DEP technology, including wireless control of AC fields and enhanced design flexibility – which led to increased throughput and high-fidelity parallel single-cell capture. Previously, we reported a unified platform for marker-free selection of CTCs. This platform accomplishes individual sequestration of CTCs into an array of reaction chambers aligned to BPEs, thus creating conditions appropriate for integrated selection,^38,47^ fluidic isolation^39^ and analysis of individual cells in parallel.

### Operating principles of the DEP methods employed

In this study, three distinct DEP methods are employed. Specifically, the 3DEP system and CF-DEP are used to characterize the dielectrophoretic response of melanoma cells and leukocytes as a function of electric field frequency, and then the performance of our DEP-BPE platform is evaluated. Therefore, we provide here a brief introduction to the operating principles of these methods.

The 3DEP system (DepTech, Uckfield, U.K.) comprises a chip with an array of cylindrical microwells, each addressed by ring-shaped electrodes embedded within its walls. A cell suspension is pipetted into the wells, and when the chip is inserted in the 3DEP instrument, distinct electric field frequencies are applied to each well. The redistribution of cells in each well is monitored by brightfield imaging. Cells are either focused to the center of the well (nDEP) or drawn to the walls (pDEP). 3DEP therefore records an ensemble (averaged) response of cells at each frequency. This frequency-dependent response is plotted as a DEP spectrum. A key point is that the distribution of responses (spread of cofs within the cell population) is lost due to averaging. The software automatically creates a mathematical fit of this data to the response curve, from which biophysical characteristics such as cell membrane capacitance and cytosolic conductivity can be derived. Since some of cellular properties are interdependent, it is better to input known values as fixed parameters. Medium properties are generally known, such as a dielectric constant of 78, and should be fixed. The mean cell radius and medium conductivity are input by the user.

In CF-DEP, the cell sample and a sheath fluid (DEP buffer) are flowed into an H-type microfluidic channel *via* two distinct inlets and exit *via* corresponding outlets. An interdigitated array of electrodes is embedded along the channel floor and oriented diagonally to the direction of flow such that cells experiencing sufficient pDEP force are redirected along the electrodes into the sheath fluid. By monitoring the cells exiting *via* each outlet, the percentage of cells experiencing pDEP can be quantified as a function of the frequency of the applied field. Since the percentage is made up of individual cell responses, CF-DEP provides the spread of cofs within the cell population.

Finally, our DEP-BPE platform is designed for single-cell capture at a single frequency, optimized for selection of a targeted cell type. The microfluidic chip comprises an array of BPEs aligned to an array of cell-sized micropockets, each leading to a picoliter-scale chamber (intended to isolate each cell for subsequent biomolecular assay). This array is addressed by parallel microfluidic channels, such that cells experiencing sufficient pDEP force are attracted to and captured (held) in the micropockets. The size of the micropocket, electric field strength, and flow rate are optimized to achieve single-cell occupancy of each pocket. Following step, the electric field is turned off, and only captured cells are transferred hydrodynamically into individual chambers. The clinical utility of this DEP-BPE chip motivates the current study, which seeks to identify optimal conditions for its application to the isolation of CMCs.

## Materials and methods

The silicone elastomer and curing agent (Sylgard 184), bovine serum albumin (BSA), and 0.25% trypsin–EDTA (1×) were purchased from Fisher Scientific (Thermo Fisher Scientific, Inc., Waltham, MA). The DMEM cell culture medium, dextrose (d-glucose), sucrose, Pluronic F-127 and 1.0 M Tris HCl stock were obtained from Sigma-Aldrich, Inc. (St. Louis, MO). The RPMI 1640 medium was purchased from American Type Culture Collection (ATCC) (Manassas, VA). All dilutions were conducted with type 1 water (18.2 MΩ cm). DEP buffer comprised 8.0% sucrose, 0.3% dextrose, and 0.1% BSA in 1.0 mM Tris buffer (pH 8.1) and was used within 72 h. DEP buffer was used in all DEP experiments performed on the 3DEP, CF-DEP, and DEP-BPE platforms. Patient-derived peripheral blood samples were obtained through the University of Iowa's Biospecimen Procurement and Molecular Epidemiology Resource Core (BioMER) programs in compliance with guidelines set by the Department of Health and Human Services to protect human subjects. The samples were collected following a protocol approved by the University of Iowa Institutional Review Board (IRB), and informed consent was obtained from all human subjects who participated. The microfluidic device design and fabrication, biological materials and blood samples, and protocols for cell spike-in experiments, fluorescence labeling, viability test (Trypan Blue staining), characterization of DEP response of melanoma cells and PBMCs by 3DEP system, calculation of DEP response of melanoma cells using CF-DEP device and calculation of melanoma cell capture efficiency in the DEP-BPE platform can be found in the ESI.†

## Results and discussion

The following subsections describe the characterization of the DEP response of relevant cell types under static (no flow) and dynamic (with flow) conditions by using 3DEP and CF-DEP, respectively. In addition, modeling of this response to extract dielectric properties of these cells is described. The information gained from these studies allowed for the identification of optimal conditions for DEP-based recovery of melanoma cells from blood. We then quantitatively evaluated the performance of our DEP-BPE device under these optimized conditions. Finally, we implement the DEP-BPE device for the isolation of CMCs from blood derived from patients with stage IV melanoma and subsequent cell classification by immunofluorescent staining.

### Characterization of melanoma cells and PBMCs under static conditions

We determined the dielectrophoretic spectra for melanoma cell lines (A375, SK-MEL-1, SK-MEL-2 and SK-MEL-28) and PBMCs separated from healthy donor and patient-derived blood samples using the 3DEP system.^49^ The conductivity of the medium was measured every time before use (around 74.5 ± 2.3 μS cm^−1^). The mean cell radius was measured by the Countess™ system before each DEP experiment. The relative cytoplasmic permittivity was determined from where the curve leveled off at high frequencies, and it is best fixed at 60. Each melanoma cell line was evaluated within 8 passages after initial receipt, and 15 trials (3 passages × 5 trials per each) were completed for each cell line. PBMCs were isolated from two types of blood samples – those obtained from healthy donors and patients diagnosed with late-stage melanoma. Patient status for mutation to the BRAF gene was recorded as V600E, G469R, or negative. One sample was not analyzed until 3 days after the blood was drawn and is included to illustrate the impact of sample age on the dielectric properties of the cells. Another sample was not utilized due to damage during shipping and is listed as “undetermined.” During the experiment, each 3DEP® microwell chip was pretreated with DEP buffer to avoid bubble formation in the chip, which increases noise and affects the result.

**Fig. 1:**
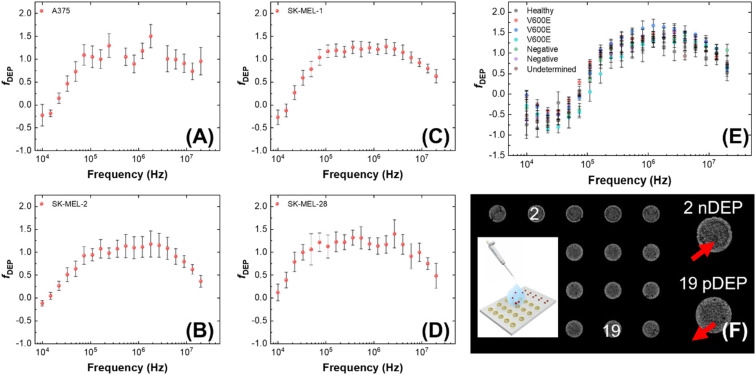
Averaged DEP spectra of A375 (A), SK-MEL-1 (C), SK-MEL-2 (B), SK-MEL-28 (D), PBMCs (E) separated from blood samples based on fifteen distinct 30 s measurements per cell line by using the 3DEP instrument. The *y*-axis is relative DEP force, in arbitrary units. PBMCs were derived from healthy controls and stage IV melanoma patients. (F) Brightfield micrograph showing a chip loaded with PBMCs, cells move towards or away (red arrows) from the electrodes at the well walls, termed pDEP and nDEP, respectively. Five separate batches of the same cell type were interrogated to ensure reproducibility.

Fig. 1 is a series of plots showing the dependence of the relative DEP force on frequency for each of several cell lines and PBMC samples. Where this force first changes sign from negative to positive is called the first crossover frequency (cof). Melanoma cells have a relatively low first cof compared to PBMCs, thus yielding the possibility to separate the melanoma cells from the background PBMCs. These plots show that melanoma cells reach the first cof under 30 kHz, while most PBMCs do not reach the cof until at least 70 kHz. This result implies that a frequency intermediate to these cofs can selectively attract melanoma cells to electric field maxima. No correlation of the cof of patient-derived PBMCs to BRAF status was observed.

Analysis of the DEP spectra further allows for the determination of cellular electrophysiological parameters.^50^ The values of melanoma cell membrane capacitance and conductance acquired by 3DEP® are listed in Table S1.† To further understand the cellular DEP response of these cell types in distinct platforms, these measured biophysical characteristics were entered into the openly available computational software MyDEP to predict the responses of each cell population over a range of applied frequencies and medium conductivities as discussed in a subsequent subsection (Modeling the dielectric properties of melanoma cells).

### Characterization of the DEP response of melanoma cells, PDXs and PBMCs under dynamic conditions

We next quantitatively determined the separation efficiency that can be achieved by DEP for melanoma cells and PBMCs using several melanoma cell lines, melanoma PDXs, and PBMCs derived from both healthy donors and patients diagnosed with advanced melanoma. Patient-derived PBMCs were included in this study to evaluate variations in PBMCs that could potentially arise due to therapy or advanced disease. The average cof for each cell type obtained by static measurements (3DEP®) provides insufficient information. Instead, the distribution of cofs within each population of cells must be ascertained.

To accomplish this goal a CF-DEP microfluidic device was utilized.^51,52^ The principle of this device is based on exertion of pDEP force by a planar array of electrodes aligned diagonally to the flow field to deflect cells laterally. A square wave is imposed over the applied AC voltage to alternate between “on” and “off” states of the electric field to prevent trapping or adhesion of cells to the electrode array. The device comprises a limited-height H-type microchannel (around 28 μm) overlaying a large array of oblique interdigitated electrodes. Cells are flowed into one inlet, while a cell-free sheath flow is flowed into the other inlet. The direction of the DEP force depends on the frequency of the external AC electric field and the dielectric properties of each cell. Cells that experience no force or nDEP will remain in the same streamline and exit the device *via* the outlet on the same side that they entered. Cells experiencing pDEP force are directed along the electrodes to the opposing outlet. Here, we carried out frequency sweeps from 5 kHz to 200 kHz for each cell line and then plotted the percentage of cells experiencing pDEP (exiting *via* the opposing outlet) as a function of frequency at a resolution of 5 kHz. Image analysis was used to count the number of cells exiting each outlet. In these experiments, about 2.5 × 10^4^ cells were analyzed in the CF-DEP device for each trial (*n* = 5 trials).

Prior to performing these frequency sweeps, we determined the appropriate voltage to be applied to the interdigitated array. We noticed that, at a fixed frequency, the applied voltage alters the number of cells exiting from each outlet. This behavior can be explained by considering that when a weak electric field is applied, even if Re[*K*(*ω*)] is high (close to 1), the resulting *F*_DEP_ is too weak to deflect cells laterally against the hydrodynamic force.^51^ To find a suitable electric field strength, we increased the applied voltage from 3 V_pp_ to 9 V_pp_ at 50 kHz, and recorded the DEP response of SK-MEL-2 cells. The total flow rate was fixed at 1.2 μL min^−1^. At 3 V_pp_ and 4 V_pp_, almost no cells exited through the opposing outlet, after increasing to 5 V_pp_, some cells (around 25%) began to be deflected to that outlet by pDEP. At 6 V_pp_ and 7 V_pp_, the fraction of cells exiting *via* the opposing outlet was stable. In contrast, increasing to 8 V_pp_ decreased this percentage, and at 9 V_pp_, some cells were captured or even lysed on the surface of the electrodes, which meant the electric field was too high to be used at this flow rate. Based on these results, we selected 6 V_pp_ for subsequent CF-DEP experiments.

**Fig. 2:**
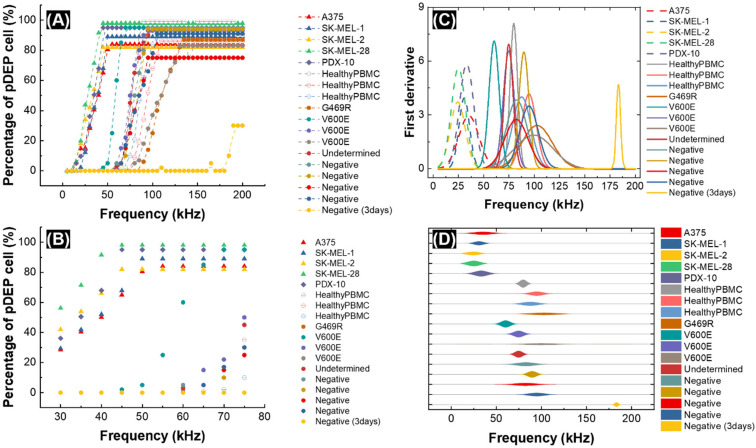
A plot of the percentage of cells exhibiting a pDEP response in CF-DEP for melanoma cell lines (triangles), a patient-derived xenograft (diamonds), and PBMCs separated from healthy donor (open circles) and patient-derived (filled circles) blood samples is shown in (A). A zoom-in plot of the results in (A) from 30 kHz to 75 kHz (B). A normal distribution fitted to the pDEP response for each cell line (C) was used to select the optimal frequency (50 kHz) for CMC isolation. The dotted lines indicate melanoma cell lines and the solid lines indicate PBMCs from healthy donor and patient blood samples. A kite diagram of cellular pDEP response is plotted as a function of frequency in (D). Applied voltage: 6 V_pp_.

Fig. 2 shows the result of CF-DEP under a frequency sweep for each cell type. In Fig. 2A, the percentage of cells exiting *via* the opposing outlet (undergoing pDEP) is plotted as a function of the applied frequency (error bars for melanoma cell samples shown in Fig. S2†). A small fraction of melanoma cells (triangles) and PDX-10 cells (diamonds) exhibit a pDEP response below 25 kHz, and most cells from these populations respond before 50 kHz. As predicted by 3DEP® analysis, PBMCs derived from healthy controls and melanoma patients require a higher applied voltage to yield a pDEP response (50–125 kHz). Notably, the patient PBMCs have a wider frequency distribution compared to the PBMCs obtained from healthy donors, and some exhibit a pDEP response at low frequency. Fig. 2B is a close-up view of the data shown in Fig. 2A over the frequency range of 30–80 kHz. This plot better illustrates the fraction of target (melanoma) and non-target (PBMC) populations that are collected by pDEP over the range of frequencies relevant to separation. For example, for all but one patient sample, melanoma cells and PBMCs are well separated at 50 kHz. Note that the percentage of cells experiencing pDEP does not reach 100% in all cases. We attribute this outcome to a fraction of dead cells present in each sample.

Fig. 2C and D show the first derivative of the data presented in Fig. 2A. In Fig. 2C, the data is fitted to a normal distribution, and the area under the curves is normalized. This derivative corresponds to the change in the percentage of cells experiencing pDEP (the fraction of cells that experience “crossover” to pDEP) at each frequency. This data shows that melanoma cells have a narrow distribution of cofs and that patient-derived PBMCs have the widest distribution of cofs. The Gaussian fit to each population was utilized to predict the probability of a pDEP response for each cell line at selected frequencies (Table S3†). Most notably, at 50 kHz, greater than 99% of all melanoma cell types will have a sufficient pDEP response to be deflected to the opposing outlet. For PBMCs at this frequency, this fraction is essentially zero in samples from healthy donors, and among the nine melanoma patients screened, was distributed broadly from less than one in a billion up to as high as 0.1%. For perspective, this means that at 50 kHz, introduction of 10^7^ patient-derived PBMCs (the approximate number of PBMCs in one milliliter of whole blood) onto the chip will yield unwanted capture of between zero and 10^3^ PBMCs.

This potential for a high degree of contamination may motivate pre-screening of patient samples to assess PBMC properties prior to selection of the separation frequency for melanoma cell capture. For example, for the individual patient sample (patient M10339, Table S3†) for which the probability of PBMC capture at 50 kHz was 0.1%, this probability decreases by two and three orders of magnitude at 45 kHz and 40 kHz, respectively. At these lower frequencies, the probability of recovery among all melanoma cell types tested ranged from 94.1–99.9% and 77.6–99.4%, respectively. Based on these results, we conclude that a shift to these lower frequencies for such a patient sample represents a reasonable compromise between capture efficiency and purity.

### Modeling the dielectric properties of melanoma cells

**Fig. 3:**
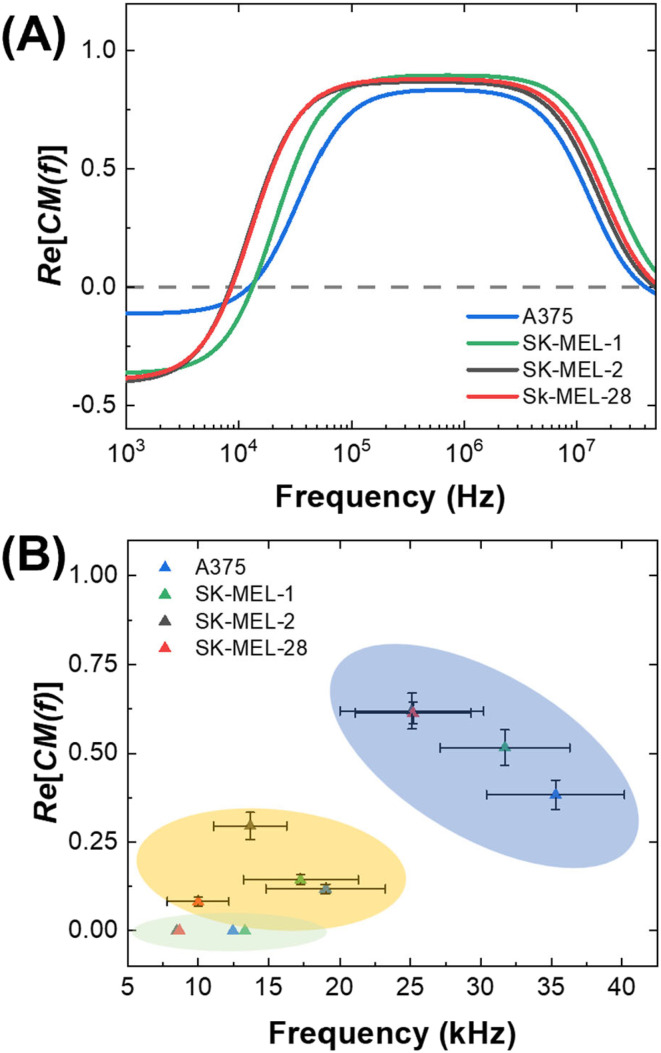
Demonstration of the shift of cof between three distinct DEP platforms. (A) Plot of DEP spectra predicted from cell properties by MyDEP. These results were used to find a static cof for melanoma cells (green area), which is plotted in (B) *versus* the CM factor corresponding to that frequency in the spectra shown in (A). Dynamic cofs are the observed transition of each cell line to a “pDEP response” in the 3DEP and CF-DEP platforms and are shown in the yellow and blue areas, respectively.

Fig. 3 illustrates how cell and medium properties can be utilized to predict DEP response and the outcome of cell separations under both static (no flow) and dynamic (flowing) conditions. Experimentally measured constants are first fed into a model of a cell as a dielectric particle, and this model predicts the DEP spectrum (Fig. 3A), which in turn, informs the calculation of DEP force and cell trajectory (Fig. 1A and B). This cell trajectory determines whether cells are observed to have an outcome (capture or direction to a specific outlet) that the user defines as an “nDEP response” or “pDEP response”. The effective cof between these experimental outcomes depends on the CM factor required to yield sufficient force to achieve them with the method employed (Fig. 3B).

In the present study, DEP spectra were predicted with MyDEP, which is a computational software programmed in Java, aimed at the study of dielectrophoretic behavior of particles, including cells, suspended in a medium.^53^ More precisely, the software can calculate and display the DEP spectrum for distinct conditions (medium dielectric constant, medium conductivity, and frequency range) given the dielectric properties, size and shape of the particle. In this study, a single-shell spherical model, which comprises a cytoplasm surrounded by a cell membrane, was used. In the presence of particles, the effective permittivity of the suspension 
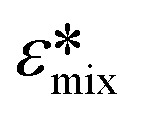
 depends on the volume fraction ∅ occupied by the particles. In MyDEP, the Hanai equation has been implemented,^53,54^ and we used 0.3 as the value of the volume fraction. As mentioned before, cofs correspond to the frequencies at which Re[*K*(*ω*)] = 0. For each electrical conductivity of the medium *σ*_m_, this value might differ. To minimize the effect from the medium, we used a consistent protocol and measured the medium conductivity every time before running DEP experiments, the value was 74.5 ± 2.3 μS cm^−1^, and we input 74.5 μS cm^−1^ for all the simulations. All the needed values for simulation by MyDEP are listed in Table S1.† A notable finding of the measurements of melanoma cell properties made in 3DEP® is that membrane conductance was observed to have a large standard deviation. To determine the effect of membrane conductance on the DEP spectrum, we used different measured values while keeping the other parameters constant, and the results showed that the effect of membrane conductance on the first cof is negligible.

Fig. 3A is a plot of the predicted DEP spectra of three melanoma cell lines in DEP buffer based on measured parameters by 3DEP® averaged over fifteen trials. These spectra are important because the response of cells at a given frequency can be correlated to the CM factor required to yield that response. Fig. 3B is the effective cof observed by 3DEP® and CF-DEP plotted against the corresponding CM factor found by MyDEP. The effective cof value shifts toward higher frequencies when measured by experimental methods (3DEP® and CF-DEP). The following section addresses this shift and discusses the concept of static and dynamic crossover frequencies.

### Definition of static and dynamic crossover frequency

Although DEP has the potential to address clinical challenges in cell isolation, a basis for predicting the DEP behavior of cells in distinct platforms has been lacking. Here, we compare the frequency at which cells undergo an observable pDEP response for each melanoma cell line on the 3DEP® and CF-DEP platforms and compare these effective cofs to the cof predicted by theory using MyDEP. The primary distinction between these platforms is the magnitude of competing forces that must be overcome by DEP. Therefore, these competing forces set the effective cof by dictating how far above zero the CM factor must be to achieve a “pDEP response” defined by displacement of the cell to a specific location.

Fig. 1A is an illustration of the forces exerted on the cells in the 3DEP® chip. Here, cells experience both gravity and buoyancy forces, and the movement of the cell through the medium driven by *F*_DEP_ is countered by *F*_drag_ (not depicted). Under conditions of fluid flow as in CF-DEP (Fig. 1B), cells experience *F*_drag_ in the *x*-direction (along fluid laminae). A key point is that the cof predicted by MyDEP, which is discussed in the preceding subsection, it is lower than that measured experimentally where there is drag.

Therefore, we define the frequency generated by MyDEP as the static cof, and the frequency measured from 3DEP® and CF-DEP as dynamic cofs. These frequencies obtained for several cell lines are listed in Table S2.† These results indicate that the frequency and CM factor required for a cell type to exhibit a “pDEP response” on each DEP platform can be predicted. Fig. 1A is a plot of the observed cof for four melanoma cell lines correlated to the CM factor (Re[*K*(*ω*)]) predicted for these same cell lines by MyDEP at those frequencies. For example, the dynamic cof measured for SK-MEL-28 by CF-DEP is 25 kHz, which is higher than its static cof and corresponds to a CM factor of about 0.62 on the DEP spectrum predicted by MyDEP. When introducing a new cell type with matched radius and shape, which scale *F*_drag_ and *F*_DEP_, a frequency capable of yielding that same CM factor would be required to reach its dynamic cof.

**Fig. 4:**
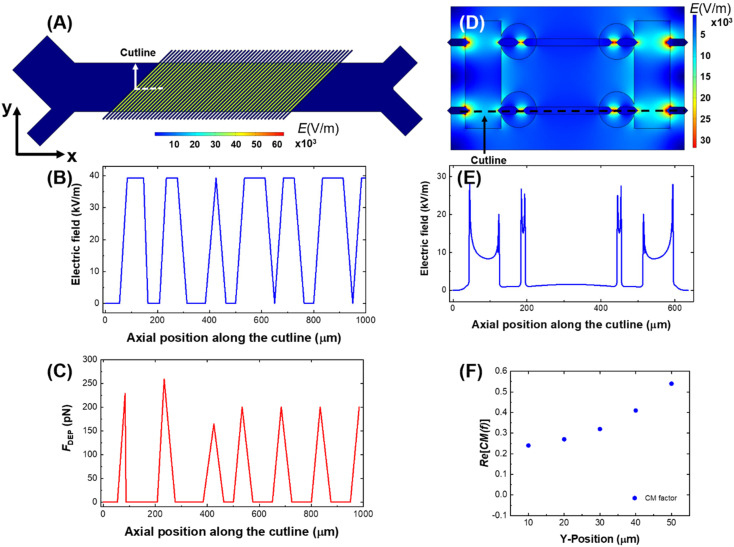
Numerical simulation of the electric field strength surface plots (A and D) and line plots (B and E) and DEP force line plots (C) in the CF-DEP device (A–C) and DEP-BPE device (D and E). Numerical simulation of CM factor required for capture of A375 cells in various *y*-positions in the DEP-BPE device, where *y* = 0 μm is defined as the channel wall in line with the opening to the micropocket (F). Line plots were taken along the cutline indicated in (A and D). The dimensions (channel width, pocket size and electrode positions and shapes) matched those of device employed in this study, and pDEP force was estimated based on a 20 μm-diameter cell at a CM factor of 1.0 for CF-DEP.

The results of a numerical simulation of the distribution of the electric field and DEP force experienced by a cell in the CF-DEP device is shown in Fig. 4A–C. Variability in local maxima in field strength and force across the electrode array is due to the limited resolution of the simulation. In CF-DEP, assuming Re[*K*(*ω*)] = 1.0, a cell with a 20 μm diameter experiences *F*_drag_ = 74 pN and *F*_DEP_ ≈ 200 pN. As a result, the cell experiences a net force of about 140 pN along the *y*-axis, leading it to the upper outlet. In the DEP-BPE device, however, the orientation of *F*_DEP_ changes as a cell flows along the channel, which makes it difficult to predict the CM factor required for cell capture. To solve this problem, we simulated cell capture in the DEP-BPE device. The conditions for the simulation were a fixed average linear flow velocity of 150 μm s^−1^ and fixed applied voltage of 4 V_pp_ for one channel (scaled down from 16 V_pp_ in a four-channel device). The properties of A375 cells are used for this model since it has the highest cof among the melanoma cell lines we evaluated and is therefore, the most difficult to capture. Finite element models of channel segments comprising two chambers on either one side of the channel or oriented across from each other were created based on the real dimensions of the DEP-BPE device. Particles representing cells were introduced into the segment from several distinct *y*-positions. In the device with chambers on just one side, when a cell is flowed along the center line of the channel (*e.g.*, the center of the cell is located 50 μm away from the wall of the channel), the simulation shows that a CM factor of at least 0.54 is required to capture the cell in the first chamber. This CM factor corresponds to 49.7 kHz on the MyDEP curve. In the DEP-BPE platform (Fig. 4D–F), cells show a range of CM factors required depending on *y*-position, with the highest CM factor required along the center-line. Results of the simulation can be found in Videos S1 and S2.†

### Quantification of capture efficiency for melanoma cells in the DEP-BPE device

We next evaluated the capture efficiency for melanoma cells at 50 kHz in the DEP-BPE platform. This capture efficiency is expected to match that predicted by our study (Table S3†) until a sufficient number of pockets are occupied, thereby preventing further capture. Two melanoma cell lines and PDX-10, were flowed into the DEP-BPE device, and their capture efficiency was quantified as the ratio of cells captured to those being introduced. The DEP-BPE device design utilized here has four parallel channels expanding from a single outlet by a bifurcation scheme, and along each of these four channels are 20 chambers, for a total of 160 chambers. The active area of the device is 7.0 mm long by 3.9 mm wide.

Capture efficiency was determined for each cell line with an experimental procedure as follows. First, melanoma cells (SK-MEL-28) tagged with dye-linked antibodies to melanoma-specific cell-surface antigens (PE-anti-MCAM and Alexa 647-anti-MCSP) were resuspended at a concentration of approx. 10^6^ cells per mL in DEP buffer followed by serial dilution to 5 × 10^4^ cells per mL. Then, into a device prefilled with DEP buffer, this sample was introduced at a constant flow rate of 100 nL min^−1^ (6 μL h^−1^) under an applied voltage of 16 V_pp_ at 50 kHz. These conditions were maintained for 25 min while the number of cells entering the device was monitored. Cells that experienced sufficient pDEP force were attracted to and captured in the pockets. Finally, the captured cells were evaluated by fluorescence microscopy to ascertain the occupancy of each chamber (*i.e.*, empty, single or multiple).

**Fig. 5:**
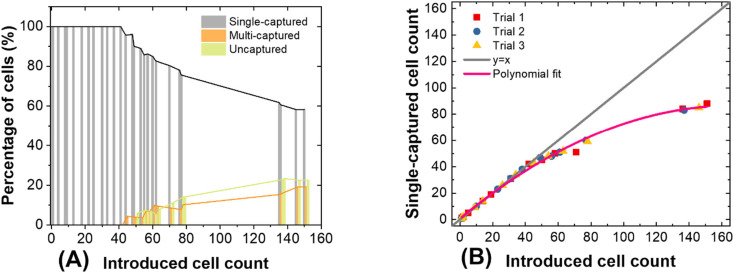
Performance of the 160-chamber DEP-BPE platform in the capture of SK-MEL-28 cells under optimized conditions. (A) Plot of the percentage of single-captured, multi-captured, and uncaptured cells as a function of the total number of cells introduced. (B) Plot of the number of cells captured individually *versus* the total number of cells introduced. Polynomial fitting was used to demonstrate saturation effect as deviation from *y* = *x*. In each trial, between 150 and 160 cells were introduced over a period of 25 min.

Fig. 5A is a plot showing the percentage of cells that underwent single capture, multiple capture, or were not captured as a function of the number of cells introduced. Initially, about 95% of SK-MEL-28 cells were captured individually, and as more cells were introduced (>40 cells), the incidence of multi-capture events increased. At >50 cells, an increasing fraction of cells were not captured. We conclude from this result that the single-cell capture efficiency decreases with device occupancy (beyond 25% occupied). Fig. 5B is a distinct representation of the same dataset – it is a plot of the number of cells captured *versus* the number introduced. This relationship is linear with unit slope at low numbers of cells, and deviates beyond 40 cells captured. This result is significant for two reasons. First, the single-cell capture efficiency fits that predicted by the normalized data for SK-MEL-28 at 50 kHz (Fig. 2C, Table S3†) when less than 40 cells are introduced. Second, the result guides the selection of array size to avoid saturation effects.

This phenomenon is not only observed in DEP technology, some other methods like hydrodynamic separation^55^ and centrifugation^22^ also encounter this problem. Kim *et al.*^56^ and Eyer *et al.*^57^ both reported around 27% single-cell occupancy can be reached, and multiple cell capture often occurred when more cells were introduced. This phenomenon is due to the decreasing probability that a cell will encounter an unoccupied chamber. Further results obtained after introducing larger numbers of cells (about 150 cells) are presented in Fig. S4.† At such high occupancy, capture efficiency is drastically reduced. However, since CMCs are very rare the number anticipated from a 7.5 mL blood sample ranges from 0 to 8042 CMCs.^42^ Where more CMCs are anticipated, an expanded platform or a smaller sample volume can be employed. For the latter strategy, CMC capture can be stopped once a threshold number of pockets are occupied or the entire sample volume has been processed, whichever occurs first.

It is worth noting that cell concentration and flow rate are important parameters that influence capture efficiency. First, cell loading density must be kept low enough to avoid cell–cell dielectric interactions, which can decrease capture efficiency.^26,58^ Second, to obtain optimal single-cell capture performance, the flow rate must be optimized at the applied voltage. In our DEP-BPE device, 100 nL min^−1^, 16 V_pp_ and 50 kHz were used for all cell types. Under these conditions, SK-MEL-28 cells yielded the highest capture efficiency (100% when less than 40 cells were introduced).

### Recovery of spiked-in melanoma cells from normal blood samples with the DEP-BPE device

To test the performance of the DEP-BPE device in the isolation of melanoma cells from blood, we carried out a spike-in study. First, 50 cultured melanoma cells (SK-MEL-28) were spiked into 10.0 μL of DEP buffer, which contained 10^7^ PBMCs mL^−1^ (10^5^ PBMCs total). This concentration corresponds to about 5000 CMCs mL^−1^ of whole blood, which is higher than what is found in a typical sample derived from a patient with metastatic melanoma (about 1 CMC mL^−1^) or even the highest CMC counts reported (about 1000 CMCs mL^−1^).^41^ We chose this high concentration to provide sufficient melanoma cells to estimate capture efficiency given the low volumetric throughput of our prototype 4-channel device. Then, this sample was introduced into the device inlet at 100 nL min^−1^, while 16 V_pp_ and 50 kHz was applied at the driving electrodes. After the full 10.0 μL sample had been flowed through the device (100 min), the captured cells were fixed and labeled on-chip (as described in the ESI†) to identify and count melanoma cells and PBMCs.

**Fig. 6:**
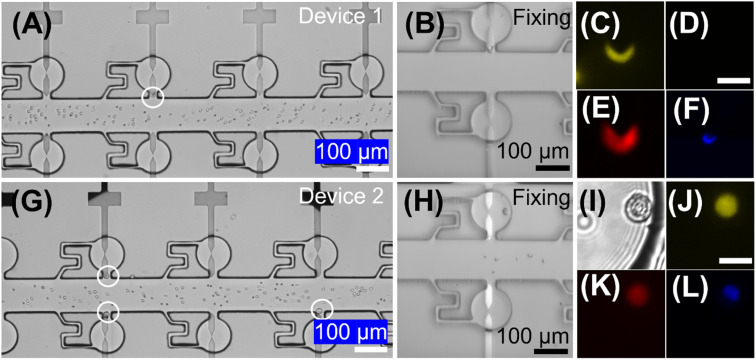
Brightfield micrographs (A, B, G, H and I) showing cells captured in the DEP-BPE platform during a spike-in experiment. SK-MEL-28 cells spiked into healthy PBMCs were introduced at 100 nL min^−1^ under an applied voltage of 16 V_pp_ at 50 kHz. After 2 h, the cells were labeled on chip. Fluorescence micrographs show captured SK-MEL-28 cells in (C and J) yellow (MCAM), (E and K) red (MCSP), (F and L) blue (Hoechst nuclear stain), and (D) green (CD45) channels. (I) Is a higher magnification brightfield image of an individually captured SK-MEL-28 cell. The scale bars in (A, B, G and H) are 100 μm and in (C–F) and (I–L) are 20 μm.

Fig. 6 is a series of brightfield and fluorescence micrographs showing a few of the captured cells in the DEP-BPE device during capture (Fig. 6A and G), cell fixation (Fig. 6B and H), and after staining (Fig. 6C–F and I–L). The recovery efficiency of SK-MEL-28 was 74%, which is lower than that predicted (∼90%) by experiments performed with pure melanoma cells at high dilution (Fig. 4B). This phenomenon can be explained by dipole–dipole interaction between the melanoma cells and PBMCs.^26^ Most of the captured melanoma cells were captured individually, and after on-chip labeling, only a few of the cells (4 and 5 in two trials) were found to be WBCs.

### Recovery of CMCs from patient-derived blood samples with the DEP-BPE device

To evaluate the feasibility of this DEP-BPE device for clinical application, we next isolated melanoma cells from blood samples (*n* = 7) obtained from patients diagnosed with stage IV melanoma. For each sample, first, the buffy coat was isolated then resuspended at a concentration of approx. 10^7^ PBMCs/100 μL in DEP buffer. Second, 12.0 μL (1.2 × 10^6^ cells) of this sample was flowed into the DEP-BPE device at 100 nL min^−1^ under an applied voltage of 16 V_pp_ at 50 kHz. Third, captured cells were fixed and labeled as described in the ESI.† Here, only Hoechst 33342 positive, MCSP positive, MCAM positive, and CD45 negative cells were considered to be CMCs, and cells having CD45 expression were considered background PBMCs. These melanoma markers were selected because it was reported that MCSP and MCAM were expressed in over 85 and 70% of primary and metastatic melanoma lesions, respectively.^59^ As a further point of reference, the distribution of the expression of MCSP and MCAM was quantified in four melanoma cell lines (Fig. S5†).

**Fig. 7:**
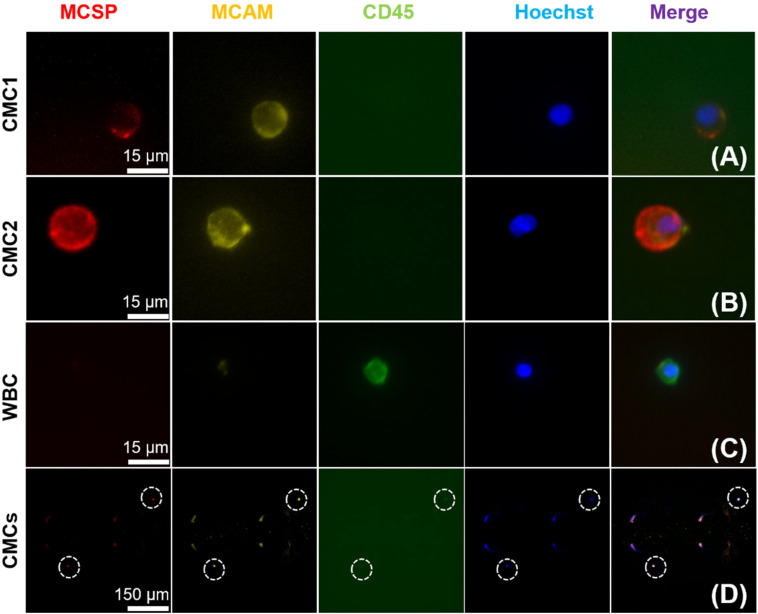
Fluorescence micrographs showing differential staining of cells isolated by the DEP-BPE platform from peripheral blood samples obtained from patients diagnosed with stage IV melanoma. Results are shown for two CMCs (CMC1 and CMC2), a WBC, and a zoomed-out image to show proximity of two CMCs in the device (white dashed circles). CMCs are positive for MCSP, MCAM, and nuclear stain (Hoescht) and negative for CD45. WBCs are positive for CD45 and nuclear stain only.

Each of these experiments resulted in a small number (2–5) of CMCs captured individually in the DEP-BPE device. These counts correspond to hundreds of CMCs mL^−1^, which is reasonable for the advanced stage of cancer in these patients. Fig. 7 is a series of fluorescence micrographs showing the result of staining for two example CMCs (close-up and zoomed out (bottom row)) and a WBC. The successful capture of CMCs suggests that the DEP-BPE platform has potential to provide access to label-free detection and individual isolation of rare cells. The volumetric throughput of this 4-channel device limited our sample to 1.2 × 10^6^ cells, which is equivalent to 10–15 μL of whole blood. However, this method is scalable by increasing the number of parallel channels as shown in our previous publication^38^ and can be further aided by on-chip pre-sorting techniques to remove the majority of unwanted cells.

The current standard of care for patients with melanoma does not include routine counting or analysis of CMCs, and where these facilities are present, they lack single-cell resolution. The methods described here, when scaled for clinical application, have the potential to provide information to guide treatment decisions, such as the level of tumor burden and molecular features that vary among tumor cells, such as those relevant to disease progression, treatment response, chemotherapeutic resistance, and invasiveness. Single-cell assays under development for the DEP-BPE platform include analysis of cytosolic enzymes, gene expression, as well as enzyme and cytokine secretion, which necessitate viable, unlabeled cells. Future clinical studies by our group will examine the correlation of such features to treatment outcomes.

## Conclusion

This paper describes the DEP-based isolation of CMCs, which lack reliable surface antigens and are extremely rare in the blood. Cells examined in this study come from three sources: melanoma cell lines, melanoma cells from a PDX, and PBMCs from both normal donors and patients with late-stage melanoma. To identify the best conditions for CMC isolation on our DEP-BPE platform, two established DEP-based separation techniques have been employed: 3DEP and CF-DEP. The dielectrophoretic responses of these cell types measured in 3DEP and CF-DEP yielded averaged DEP spectra and the distribution of cofs within each population, respectively. A key finding is that the distribution of dielectric properties of PBMCs is more broadly varied in and among late-stage patients *versus* healthy donors. Based on these measurements, the probability of melanoma cell capture and unwanted isolation of contaminating leukocytes was calculated (Table S3†). Furthermore, an open-source software, MyDEP, was used to compare and study the shift of effective cof between DEP platforms. Results of this evaluation along with the results of numerical simulations reveal the CM factor required to achieve separation in each DEP-based method and the corresponding frequency required to achieve selective capture of CMCs.

Following this evaluation, 16 V_pp_ at 50 kHz was selected as the best frequency (for the highest capture efficiency of CMCs and low contamination of WBCs) to be applied in DEP-BPE device. When these optimized conditions were employed, excellent single-cell capture (100%) was achieved when less than 40 cells were introduced into this 160-chamber device, which corresponds to 25% occupancy of the total number of chambers. Isolation of melanoma cells from contrived (spike-in) samples yielded a capture efficiency of 74%, which is lower than that achieved for pure melanoma cells due to a high concentration of PBMCs. Finally, isolation of CMCs from clinical samples with the DEP-BPE platform was demonstrated. Ongoing research in our group is aimed at scale-up and increased volume throughput of the DEP-BPE platform, which will allow direct comparison of this DEP-based approach to prevailing methods for CMC isolation from clinical samples. Molecular assays that will leverage the single-cell isolation provided by this platform will be reported in due course. In conclusion, we anticipate the results of this quantitative study will inform the design of the next generation of DEP-based platforms for CMC isolation and analysis.

## Conflicts of interest

There are no conflicts to declare.

## Supplementary Material

LC-023-D2LC01113A-s001
