# Supplementary material for: Quantification of capture efficiency, purity, and single-cell isolation in the recovery of circulating melanoma cells from peripheral blood by dielectrophoresis
Source: Lab Chip. 2023 Apr 7;23(11):2586–600. doi: 10.1039/d2lc01113a (PMC10228177; doi:10.1039/d2lc01113a)
Supplement: LC-023-D2LC01113A-s001 [file LC-023-D2LC01113A-s001.pdf]

Electronic Supplementary Material (ESI) for Lab on a Chip.
This journal is © The Royal Society of Chemistry 2023

Supporting information

Quantification of Capture Efficiency, Purity, and Single-Cell Isolation in the Recovery of Circulating Melanoma Cells from Peripheral Blood by Dielectrophoresis

Han Chen[1], Sommer Y. Osman[1], Devon L. Moose[2], Marion Vanneste[2], Jared L. Anderson[1], Michael D. Henry[2,3, 4], Robbyn K. Anand[1]*

[1]Department of Chemistry, Iowa State University, Ames, Iowa 50011, United States

Departments of [2]Molecular Physiology and Biophysics and [3]Pathology, Urology and Radiation Oncology, [4]Holden Comprehensive Cancer Center, University of Iowa, Iowa City, IA 52242

*To whom correspondence should be addressed

E-mail: rkanand@iastate.edu

This document contains the materials and methods of

1. Microfluidic device design and fabrication
2. Biological material and blood
3. Cell spike-in experiment
4. Fluorescent labeling
5. Characterize the DEP response of melanoma cells and PBMCs using 3DEP system
6. Quantify the DEP response of melanoma cells using continuous-flow DEP device
7. Quantify melanoma cells capture efficiency based on DEP-BPE

Materials and Methods

1. Microfluidic device design and fabrication

The dimensions for two device designs are described here. For the continuous-flow DEP microfluidic chip, the width and length of the microfluidic channel were 2 mm and 13 mm, respectively, and the channel had a height of 25 μm, the width of the sample inlet and buffer inlet was 250 μm and 1.75 mm, respectively. The interdigitated electrode array contained 50 electrodes, 50 μm in width with a gap of 50 μm.

For the DEP-BPE, the 4 parallel microchannels with each being 7 mm long × 110 μm wide × 25 μm tall were arranged in parallel and separated by 590 μm. Each channel had 20 chambers extruded at each side (160 chambers). Each chamber was 110 μm diameter and edge-to-edge distance of two adjacent chambers was 330 μm. The microchannels were interconnected to a common inlet and outlet by a bifurcation (branching) scheme. A leak channel (7 μm wide) was affixed to each chamber to make a fluidic connection to the main channel.

The features were designed using AutoCAD (Autodesk, San Rafael, CA) and written on a chrome mask. Soft-lithography was used to pattern the structures of the continuous-flow DEP and DEP-BPE device. Negative photoresist (SU-8 2025, MicroChem Corp., MA, USA) was spin-coated onto a four-inch silicon wafer and was subsequently subjected to several physicochemical processes, namely soft baking, UV light exposure, post-exposure baking, development, and hard baking. As a result, the SU-8 master template was formed on a silicon wafer with a thickness of

25 μm to the proposed design. Next, for replica molding, an instant barrier was made by wrapping the master silicon wafer in aluminum foil. A 10:1 volumetric mixture of PDMS (Sylgard 184, Dow Corning Corp., MI, USA) and a curing agent were then poured onto the master wafer. After degassing the polymer mixture, the master wafer overspread with clear PDMS was cured in room temperature, then the PDMS replica was removed from the master wafer and perforated at the channel inlet and outlet using a punch. The punch was used 1 mm for inlet and 2 mm for outlet.

The DEP electrodes were fabricated on a four-inch glass wafer using a conventional photolithography process. Photoresist (PR, AZ4620) was spin-coated at 3000 rpm for 35 seconds to achieve approximately a thickness of 7 μm, exposed under UV light and developed to define the electrode patterns. AZ developer, gold etchant and chrome etchant were used successively to define the electrodes, and NMP was used to remove the patterned PR. Lastly, the PDMS replica was assembled with fabricated electrode patterns onto the glass wafer after air plasma treatment using a plasma cleaner and was stored overnight in an oven (65 °C) to ensure permanent bonding. Each device was pretreated with 3 μM pluronic F-127 solution and incubated at 4 °C overnight to effectively inhibit cell adhesion on PDMS and electrodes. Each sample was run using a new disposable polydimethylsiloxane (PDMS) chip to prevent contamination.

2. Biological material and blood

Four melanoma cancer cell lines, A375 (V600E, homozygous), SK-MEL-1 (V600E, heterozygous), SK-MEL-2 (WT) and SK-MEL-28 (V600E, homozygous) (American Type Culture Collection, Manassas, VA) and a cell line (PDX-10) derived from a patient-derived melanoma xenograft at the University of Iowa were used to characterize DEP response. They were cultured in DMEM media with 1% non-essential amino acid and 10% fetal bovine serum (FBS) supplementation at 37 °C and 5% CO_2 in a humidified environment. In preparation of DEP experiments, A375, SK-MEL-2 and SK-MEL-28 were detached from culture flask using 0.25% Trypsin-EDTA (1x), followed by pelleting by centrifugation (1100 rpm, 5 min), washed with culture media or DPBS and resuspended in working buffer. The dielectric characteristics of the cell lines were measured within 8 passages after initial receipt.

Whole human blood, individually drawn from healthy donors, was provided by University of Iowa. Each 4-mL draw was collected into one Strek tube containing K_3EDTA anticoagulant, stored at 4 °C before shipping, and used right after receiving. PBMCs were processed using density gradient separation, whole blood cells were first floated on Ficoll-Pague (GE Henlthcare Bio-Sciences Corp., Uppsala, Sweden) and then centrifuged for 40 min at 400 xg, followed by careful isolation of the buffy coat. The isolated buffy coat was washed and diluted in DPBS to remove residual Ficoll-Pague. The final step was an additional centrifugation for 10 min at 100 xg to reduce contamination of the platelets. Each melanoma cell line and PBMC viability was evaluated with a trypan blue. The cell sample was diluted in trypan blue (1:1 ratio) and incubated for 2 min. 20 μL of the sample was then transferred into the cell counting chamber slide, then the slide was inserted into Countess™. Viable cells remain unstained and non-viable turn blue.

Patients with Stage IV metastatic cancer were recruited according to the protocol approved by the University of Iowa's Institutional Review Board. PDX-10 samples were obtained from University of Iowa.

3. Cell spike-in experiment

The number of melanoma cells spiked in will be controlled either by serial dilution (for spike in of 100-1000 cells) or by spiking in from a capillary (for <100 cells). The capillary spike-in experiments were followed as Zhao's method. (1)

Specifically, for capillary cell spike-in experiment, the glass capillary tubes (0.4 mm I.D. x 75 mm) obtained from Drummond Scientific (Broomall, PA) were placed in a vacuum desiccator jar along with a few drops of tridecafluoro-1,1,2,2-tetrahydrooctyl-1-trichlorosilane (TDF-Silane) in an open vial and maintained in a vacuum overnight. After surface modification, cell suspension was siphoned into a glass capillary and cells within the capillary were counted manually. To eject cells, a disposable positive-displacement pipet tip (Microman CP-25) from Gilson Medical Electronics (Middleton, WI) was slipped onto the capillary initially without the piston, and then the piston was inserted into the capillary tip at a constant speed. All the capillary spike-in experiments were finished within 3 minutes.

Serial dilution was used when 100-1000 tumor cells was spiked into PBMCs, when this method was chosen, the concentration of the cells was supposed to be exactly same as in the remaining solution. Typically, a concentrated cell suspension is pipetted into a diluent, and to reach the concentration level of rare cells, the mixture is then subjected to a succession of serial dilutions. The cell concentration is calibrated by sampling a small volume at some point of the dilution cascade, and the cells within were counted by Countess™.

4. Fluorescent labeling

To identify the cancer cells and PBMCs, immunofluorescence staining, and fluorescent microscopic observation were performed. Two of the antibodies are selected for positive identification of melanoma cells, anti-human MCAM (CD146) and anti-human melanoma chondroitin sulfate proteoglycan (MCSP), they are labeled with phycoerythrin and Alexa 647, respectively. There is a third antibody (Alexa 488 anti-human CD45) that bind only to a white blood cell marker (CD45, leukocyte common antigen). All antibodies target surface epitopes, such that membrane permeabilization is not necessary for access to each protein. In addition, Hoechst 33342 was used to stain the nucleus.

The positive identification of melanoma cells was used and described herein. All four melanoma cell lines were labeled with Alexa Fluor® 647 mouse anti-chondroitin sulfate (BD Pharmingen) and PE anti-human CD146 antibody (BioLegend; San Diego, CA). Specifically, for labeling 10^6 cells, 5.0 μL of the appropriate antibody solution was diluted to 100 μL in cell labeling buffer, which comprises dye-linked antibodies diluted in blocking buffer – a buffered saline solution containing FBS to help minimizing non-specific binding of antibodies (1x PBS with 10% FBS). Specifically, the 7.0 μL of this labeling buffer was vortexed for 1 min to disperse antibodies and to break up aggregates. Then the mixture was centrifuged at 14000 rpm (4.0 °C, 10 min), 5.0 μL of this solution was transferred and diluted in cell labeling buffer to get totally 100 μL cell labeling buffer, the leftover 2.0 μL was discarded. The cell pellets were washed with 1X PBS buffer or DEP buffer three times before suspending at 1.0 x 10^6 cells/mL.

For clinical samples, 1.50 to 2.00 mL of blood were transferred to a 15-mL polypropylene conical centrifuge tube (Beckton Dickinson, Franklin Lakes, NJ, USA). Prior to performing the

analysis of CMCs in the DEP-BPE device, the efficiency of the staining method for CMC identification was determined. In this study, CD146+ , MCSP+ and CD45- cells were determined as melanoma cells. (2) The efficiency for each labeling molecules were shown in Figure S5.

For cell captured by DEP-BPE, fresh DEP buffer was added to wash the channels, followed by cell fixation with 4% paraformaldehyde (PFA) for 10 min at room temperature. Third, the DEP AC voltage was turned off, 1×PBS buffer was flowed to rinse the device for 30 min, and labeling buffer (anti-CD45 Alexa 488, anti-MCAM PE, and anti-MCSP Alexa 647 diluted in blocking buffer) was introduced and incubated in dark for 30 min. Fourth, 30 μL 1X PBS was added to rinse for another 30 min (flow rate: 500 nL/min), followed by adding Hoechst 33342 into the leftover 15 μL PBS and changing flow rate to 100 nL/min for another 15 min.

Image analysis algorithms incorporated in MATLAB (MathWorks) allowed the accurate quantification of both cell size and fluorescence intensities. Image acquisition (stored as a TIFF stack) was analyzed for various cellular attributes, including cell area, cell size, and fluorescence intensity. Data obtained from the processing algorithm were then plotted as merged image, then the MCSP and MCAM expression percentage could be reached by compare brightfield and fluorescent images.

5. Characterize the DEP response of melanoma cells and PBMCs using 3DEP system

The working buffer ("DEP buffer") was made right before each experiment, it is comprised of 8.0% sucrose, 0.3% dextrose, and 0.1% BSA in 1.0 mM Tris buffer (pH 8.1) and used within 72 h. DEP spectral measurement was performed with unlabeled cells on a 3DEP dielectrophoretic analyzer (DepTech, Uckfield, U.K.), experimental methods and data analysis followed previously published procedures. (3, 4)

Briefly, approximately 80 μL of cell suspension was injected into the DEP well chip (3DEP 806), and a cover glass placed on top to avoid the formation of a meniscus, due to surface tension, which could interfere with the measurement of light intensity changes. The chip was mounted on a camera setup where a light beam is directed from the top of the chip, and the door was closed to prevent interference from ambient room lighting. A recording interval was set to 30 s at 10 V_{pp},

with data collected over 20 points between 1 kHz and 45 MHz. This procedure was repeated for 15 distinct samples (for cultured cells, 5 samples from each of 3 culture flasks).

The intensity of light changes depending on the movement of the cells by the DEP force. Light intensity, in each microwell, was measured for 30 s sweeping a frequency range from 1 kHz to 45 MHz. The light intensity vs frequency spectrum generated is fitted by the 3DEP software to a core-shell model using an iterative least square method to yield values of membrane and cytoplasmic conductivity as well as specific membrane capacitance, $c = \varepsilon_{mem}/d$. 3DEP light intensity bands from 4 to 9 only, from the platform spectrum output, were selected for each experiment to be fitted, resulting in a better DEP spectrum with Pearson correlation coefficient, $R > 0.95$. Cells were measured immediately (< 20 min) after being suspended into the DEP media to minimize artifacts due to cell stress by the non-physiological ion composition of the medium. While cells can respond rapidly to their environment, the DEP response of cells is stable over this time period.

6. Quantify the DEP response of melanoma cells using continuous-flow DEP device

The experimental test protocol was established as follows: (1) prior to all experiments, the channel, syringe and tubes were coated with 3 μM Pluronic solution overnight to reduce cell adhesion to the microchannel and electrode surfaces. The channel was washed using the DEP buffer for 10 minutes. (2) Cell samples were injected into the device from the sample inlet. DEP buffer was injected into the buffer inlet through tubes connected to a syringe pump. Sample flow rate and buffer flow rate were fixed at 200 nL/min and 1 μL/min (5:1 sheath to sample flow ratio), respectively. (3) A waveform generator, connected to the DEP device via driving electrodes, was used to apply AC voltage. The AC field was alternated between on and off. (4) Experimental results were observed using the Nikon AZ100 fitted with an Andor Zyla 5.5 sCMOS camera. Each experiment was performed to process at least a total volume of 120 μL.

To quantify the cell response for the entire frequency range (5-200 kHz) at resolution as 5 kHz, the cell concentration was maintained at 2×10^6 cells/ mL, the flow rate of cell solution was fixed at 200 nL/min, each frequency was maintained as least 1 min and frequency was sweeping

from 5 kHz to 200 kHz then back to 5 kHz, the total number of examined cells was around 2.5×10^4 for each experiment.

To test the high throughput of DEP-FFF, a different cell concentration was made and tested. The cell concentration was increased to 5×10^6 to 1×10^7 cells/ mL, the other experimental parameter was set as the same. However, when high concentration was used, the aggregation of cells was observed and the isolation efficiency was decreased.

7. Quantify melanoma cells capture efficiency based on the DEP-BPE platform

All the devices were designed to operate such that the largest cells (SK-MEL-28) solutions were flowed through the main channel, from which they were attracted to and captured at BPE tips (positive DEP, pDEP). Prior to DEP experiments, all devices were treated with DEP buffer containing 3.0 μM Pluronic solution overnight, followed by rinsing with DEP buffer for 20 min. Device operation was accomplished by the application of an AC voltage at coplanar driving electrodes at each side of the BPE array using a Tektronix AFG2011C waveform generator (Tektro-nix, Beaverton, OR) and Trek model 2205 amplifier (Trek, Lockport, NY). The AC frequency was maintained at 50 kHz. A Nikon Eclipse Ti inverted microscope and Nikon AZ-100 microscope (Nikon, Tokyo, Japan) were utilized to image cells to obtain fluorescence and bright field images, respectively.

Previously, our group has developed a protocol for single-cell capture in the parallel-channel device. (5) Briefly, when there is sufficient drag force competing with the DEP capture force, the pearl-chaining of cells can be prevented, and the single-cell capture efficiency could be increased. In our experiments, the flowing rate and the AC voltage were also tested to get the best performance. Finally, the volumetric flow rate was fixed at 100 nL/min and the voltage is fixed at 20 V_{pp}. To quantify capture efficiency for melanoma cells in the DEP-BPE device in the absence of PBMCs, melanoma cells (A375, SK-MEL-28 and PDX-10) were prepared at 10^6 cells/ mL in DEP buffer followed by serial dilution such that injection of 3 μL results in 150 cells. The cells that flowed into the microfluidic device was recorded by taking the image as 15 fps, then analyzed both manually and by MATLAB.

To quantify the incidence of unwanted PBMC capture in the DEP-BPE device. The PBMCs was re-suspended at a concentration of approx. 10^7 cells/100 µL in DEP buffer followed by injection of 20 µL (around 2×10^6 cells) into the DEP-BPE device under the same conditions employed for melanoma cells. The fraction captured will be quantified by counting the pockets occupied by cells.

Table S1. The cellular dielectric properties for four melanoma cell lines.

Cell type	A375	SK-MEL-1	SK-MEL-2	SK-MEL-28
**Membrane capacitance (mF/m^2)**	11.4 ± 1.8	15.3 ± 1.3	22.1 ± 2.5	20.6 ± 2.7
**Membrane conductance (S/m^2)**	702	193	118	131
**Radius (measured by Countess™, µm)**	7.71	7.70	8.52	8.90
**Cytoplasm relative permittivity**	60	60	60	60
**Cytoplasm conductivity (S/m)**	0.14	0.24	0.17	0.19
**Medium Permittivity**	78	78	78	78

Table S2. Static *cof* and dynamic *cof* revealed by different DEP platforms.

Cell type	Static *cof* (kHz)	Platform	Dynamic *cof* (kHz)	Platform	Dynamic *cof* (kHz)	Platform
A375	12.46	MyDEP	19.01	3DEP	35.3	Continuous-flow DEP
SK-MEL-1	13.31	MyDEP	17.26	3DEP	31.7	Continuous-flow DEP
SK-MEL-2	8.49	MyDEP	13.55	3DEP	25.1	Continuous-flow DEP
SK-MEL-28	8.67	MyDEP	9.7	3DEP	25.2	Continuous-flow DEP

Table S3. Physical properties, diagnostic status, and probabilities at selected applied frequencies of four melanoma cell lines, PDX cell, PBMCs separated from healthy donor and melanoma patients. Status indicates mutation to the BRAF gene (if known) for each patient.

Cell type	Tissue type	R (μm)	F_{co} (kHz) $\mu \pm \sigma$	Method	Gender	Status	Probability at 40 kHz (%)	Probability at 45 kHz (%)	Probability at 50 kHz (%)	Probability at 55 kHz (%)	Probability at 60 kHz (%)
A375	Melanoma	7.71	35.3 ± 6.2	Continuous-flow DEP			77.6	94.1	99.1	99.9	99.9
SK-MEL-1	Melanoma	7.70	31.7 ± 5.5	Continuous-flow DEP			93.4	99.2	99.9	99.9	99.9
SK-MEL-2	Melanoma	8.52	25.1 ± 5.9	Continuous-flow DEP			99.4	99.9	99.9	99.9	99.9
SK-MEL-28	Melanoma	8.90	25.2 ± 5.8	Continuous-flow DEP			99.4	99.9	99.9	99.9	99.9
PDX-10	PDX	7.98	33.1 ± 6.0	Continuous-flow DEP			87.5	97.6	99.7	99.9	99.9
Healthy	Blood	4.65	80.1 ± 2.7	Continuous-flow DEP			0	0	0	7^10^{-8}	48.7
Healthy	Blood	4.64	95.0 ± 4.5	Continuous-flow DEP			0	0	0	3.1^10^{-3}	36.9
Healthy	Blood	4.98	86.2 ± 4.7	Continuous-flow DEP			0	0	6.7^10^{-6}	1.6^10^{-2}	12.4
M10308	Blood	4.97	109.6 ± 9.8	Continuous-flow DEP	Male	G469R	6.1^10^{-5}	2.2^10^{-3}	5.9^10^{-2}	1.3	20.8
M10084	Blood	5.05	60.6 ± 2.6	Continuous-flow DEP	Female	V600E	0	9^10^{-8}	2.3^10^{-3}	1.6	40.9
M10297	Blood	5.04	75.1 ± 3.3	Continuous-flow DEP	Male	V600E	0	0	1.4^10^{-7}	5.6^10^{-3}	23.7
M10339	Blood	4.65	109.2 ± 9.9	Continuous-flow DEP	Female	V600E	1.4^10^{-4}	4.4^10^{-3}	0.1	2.2	33.5
M10347	Blood	4.90	75.0 ± 3.6	Continuous-flow DEP	Female	Unknown	0	0	1.9^10^{-6}	1.4^10^{-2}	15.4
M10329	Blood	4.51	83.0 ± 5.5	Continuous-flow DEP	Female	Negative	0	2.4^10^{-6}	9.8^10^{-4}	0.2	14.5
M10156	Blood	5.03	90.0 ± 4.2	Continuous-flow DEP	Male	Negative	0	0	8.3^10^{-8}	3.9^10^{-3}	45.7
M10337	Blood	5.01	82.4 ± 4.9	Continuous-flow DEP	Male	Negative	0	3.3^10^{-8}	7.4^10^{-5}	5.7^10^{-2}	15.3
M10341	Blood	5.04	95 ± 4.8	Continuous-flow DEP	Male	Negative	0	0	3.5^10^{-7}	3.9^10^{-3}	15.4

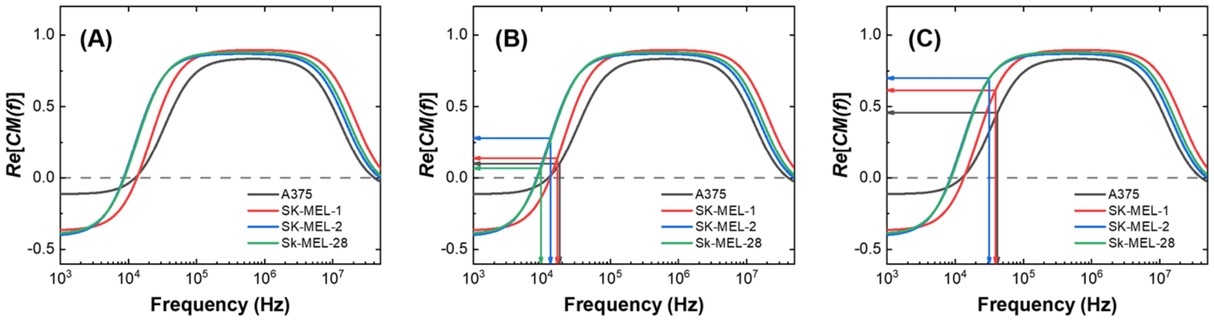

Figure S1. Prediction of DEP spectrum for four melanoma cell lines on MyDEP software (A), based on the spectrum, located the crossover frequency generated from 3DEP (B) and continuous-flow DEP (C).

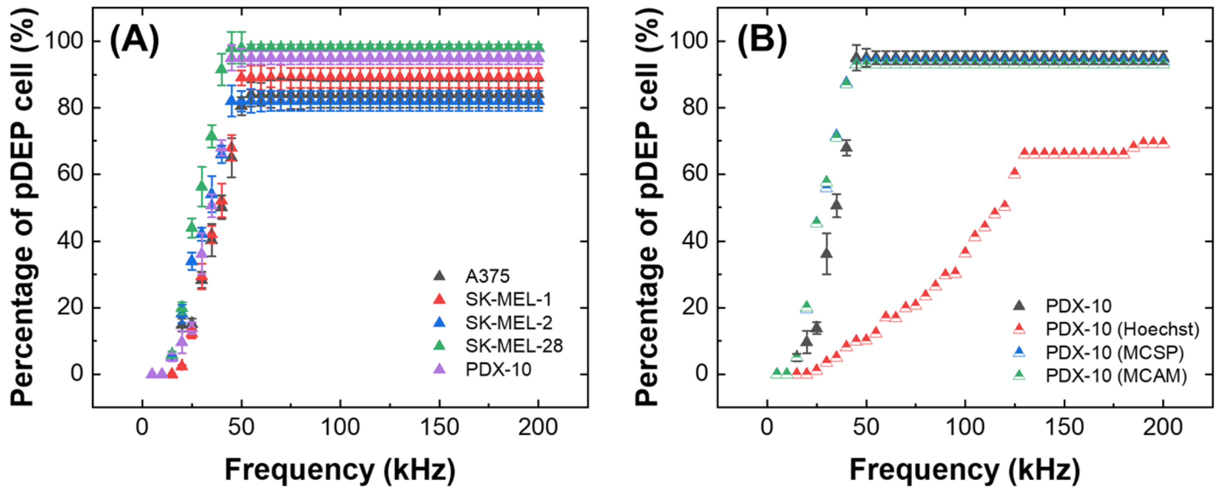

Figure S2. DEP responses of each melanoma cell line and PDX-10 were quantitatively measured by continuous-flow DEP as shown in (A). Immuno-labeling effect was evaluated by pre-labelling PDX-10 cell with each staining molecules, nuclei stain has a big effect on DEP response, as shown in (B).

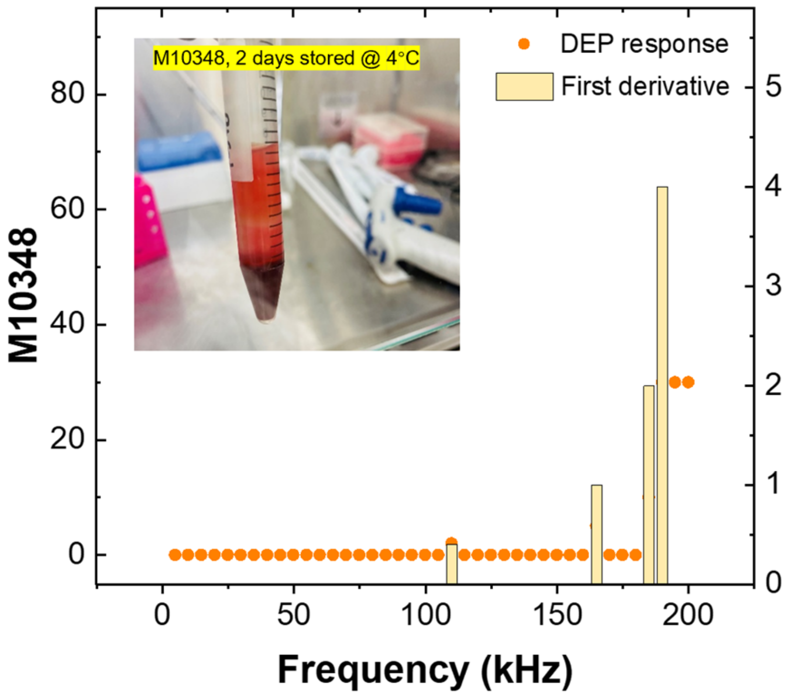

Figure S3. DEP response measurement was taken when blood sample was stored at room temperature for 3 days, the cell diameter measured was 6 µm, which could be reasonable considered as red blood contamination.

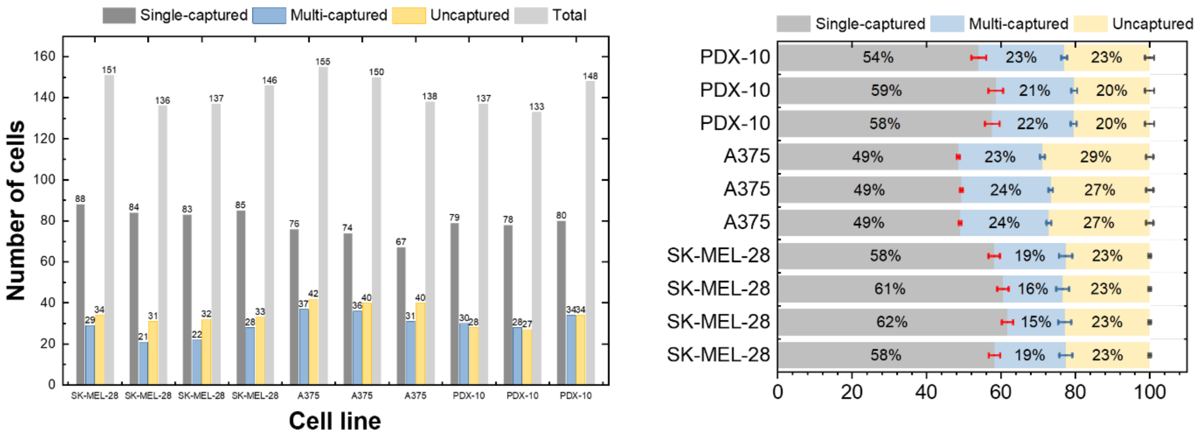

Figure S4. Capture efficiency of SK-MEL-28, A375 and PDX-10 cells on DEP-BPE when saturation effect happens.

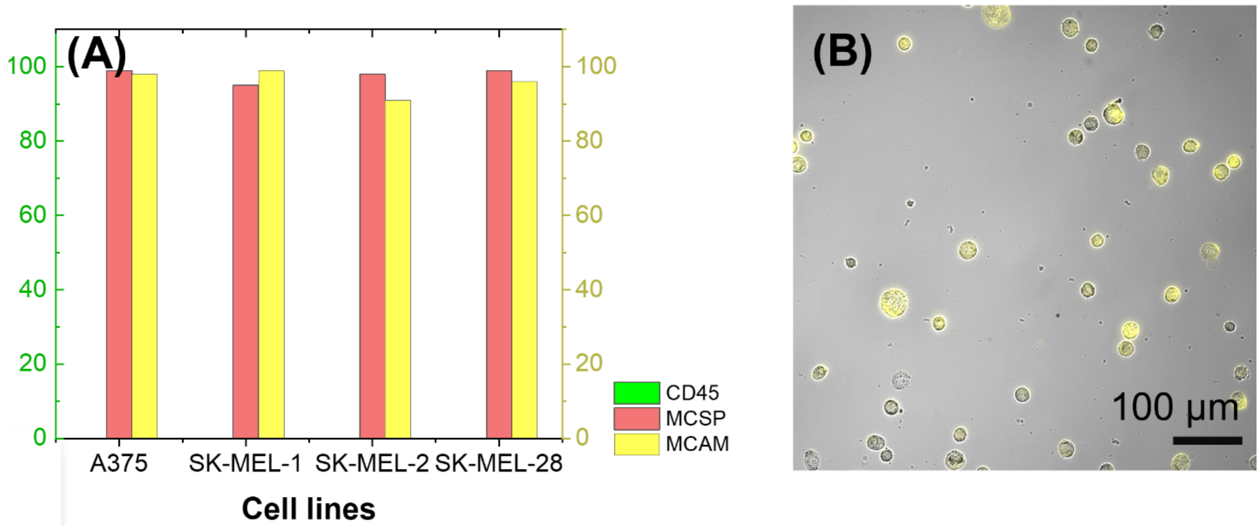

Figure S5. Labeling efficiency of four different melanoma cells, the cell viability was measured before and after immunofluorescent labeling (both > 98%). (A) shows most of the melanoma cells having expression for both MCAM and MCSP, none of them has expression of CD45. (B) shows one of the overlapping images to process the counting.

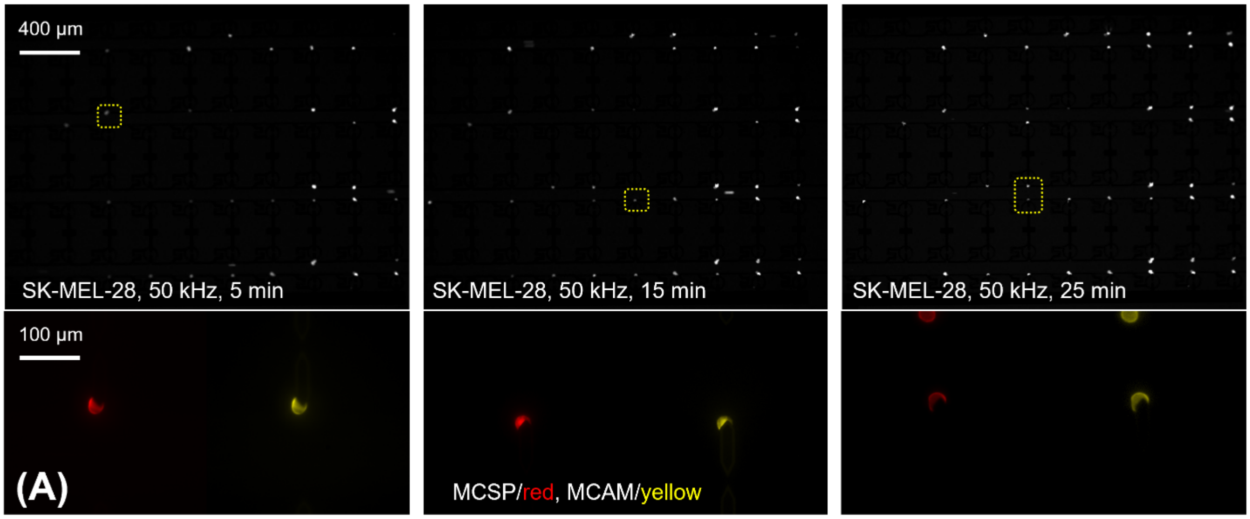

Figure S6. Fluorescence micrographs of SK-MEL-28 cells captured at 16 V_{pp} and 50 kHz in the DEP-BPE device at t = 5, 15, and 25 min after their introduction into the device. Cells were pre-labeled with Alexa 647-anti-MCSP and PE-anti-MCAM.

References

1. Y. Zhao, P. G. Schiro, J. S. Kuo, L. Ng, D. T. Chiu, Method for the accurate preparation of cell-spiking standards. *Anal. Chem.* 2009, **81**, 1285-1290.
2. C. Rao *et al.*, Circulating melanoma cells and survival in metastatic melanoma. *Int. J. Oncol.* 2011, **38**, 755-760.
3. E. A. Henslee *et al.* Rhythmic potassium transport regulates the circadian clock in human red blood cells. *Nat. Commun.* 2017, **8**, 1978.
4. K. F. Hoettges *et al.* Ten–Second Electrophysiology: Evaluation of the 3DEP Platform for high-speed, high-accuracy cell analysis. *Sci. Rep.* 2019, **9**, 19153.
5. M. Li, R. K. Anand, Integration of marker-free selection of single cells at a wireless electrode array with parallel fluidic isolation and electrical lysis. *Chem. Sci.* 2019, **10**, 1506-1513.
